# Women’s perspectives regarding in vitro fertilization as a conception option at the primary healthcare level in Ekiti State

**DOI:** 10.1007/s43999-026-00093-5

**Published:** 2026-06-11

**Authors:** Risikat Idowu Fadare, Victor Chisom Ogbujiagba, Cecilia Bukola Bello, Gbemisola Bolanle Ogbeye, Oluwafunmilayo Esther Fadare

**Affiliations:** 1 Faculty of Nursing Science, College of Medicine and Health Sciences, Afe Babalola University, Ado-Ekiti, Ekiti State Nigeria; 2 Department of Nursing, Federal University, Oye-Ekiti, Ekiti State Nigeria; 3 Medical Sdec, Acute Medicine, Wexham Park Hospital, Slough, UK

**Keywords:** In vitro fertilization, Knowledge, Perception, Attitude, Women of childbearing age

## Abstract

**Introduction:**

Infertility remains a significant reproductive health challenge globally. In vitro fertilization (IVF), the most widely used form of assisted reproductive technology (ART), presents an opportunity for overcoming childlessness. Knowledge, perception and attitudes toward IVF remain underexplored, especially in rural settings in Nigeria. This study assessed these aspects among women of childbearing age, especially at the primary healthcare level.

**Methods:**

This study employed a descriptive cross-sectional survey conducted among 222 women of childbearing age, selected from health centers in Ado Ekiti, Ekiti State, via simple random sampling. Data collected through questionnaires were analyzed using SPSS version 26, applying both descriptive and inferential statistics. The significance level was set at *p* < 0.05.

**Results:**

Most respondents (63.1%) were aged 20–29 years, with 55.9% employed and 60.8% identifying as Yoruba. Their knowledge of IVF was high, with an average score of 3.06. Overall perception of IVF was favorable (mean = 2.89), and attitudes towards IVF were positive (mean = 3.08). Moderate, statistically significant positive correlation were observed between knowledge and perception (*r* = 0.520, *p* < 0.05) and between knowledge and attitude (*r* = 0.606, *p* < 0.05).

**Conclusion:**

Women of childbearing age in Ado Ekiti exhibited high awareness, positive perceptions, and favorable attitudes toward in vitro fertilization. However, cost, accessibility, and emotional concerns remain major obstacles. It was recommended to advocate to government and stakeholders for affordable, accessible in vitro fertilization services to increase uptake and acceptance.

## Introduction

Infertility is a major global health concern, particularly in Sub-Saharan Africa, where cultural, social, and psychological expectations place a high value on childbearing within marriage. Estimates suggest that infertility affects between 13 and 17% of couples in the region, with some countries reporting rates as high as 32% [1]. In response, advances in reproductive technologies have enabled alternative methods of conception.

IVF involves fertilizing an egg with sperm outside the body and transferring the embryo to the uterus [2]. Other alternative reproduction procedures include intrauterine insemination (IUI) which is less invasive, zygote intrafallopian transfer (ZIFT), gamete intrafallopian transfer (GIFT), cryopreservation of eggs, embryo adoption (achieving pregnancy without using personal genetic material), invitro maturation (IVM), intravaginal culture (IVC), intracytoplasmic sperm injection (ICSI) (1), surrogacy, (where another woman carries the baby for the intending parents). In vitro fertilization is the most common intervention for assisted reproduction [3]. The procedure is typically recommended after repeated failed attempts at natural conception, offering hope to individuals and couples struggling with infertility.

Since the first successful IVF birth in 1978, the technique has evolved significantly and now accounts for 1–3% of all births annually in the U.S., Europe, and China [4]. In Nigeria, where the first IVF baby was born in 1989, the practice has gradually gained ground across the private and public health sectors [5]. The success rate was reportedly 20–50%. However, IVF births still account for only 1% of total annual births in Nigeria [6].

Regional variations exist in the availability, knowledge, perception and utilization of IVF. The significant urban-rural disparities are primarily driven by differences in education, access to information, and infrastructure. While urban areas benefit from greater access to information and resources, awareness is often lower in rural areas due to limited access to information sources like the media, fewer health professionals, and fewer IVF clinics. In the United States of America, for instance, a very large percentage of fertility clinics are situated in high-income areas. At the same time, they are scarce in low- to middle-income areas. This accounted for the low use of IVF in certain regions in the USA [7]. Rural areas face significant barriers ranging from geographical location, lesser educational attainment, poverty, strong socio-cultural and religious beliefs, which are attributable to misconceptions or low awareness about IVF [7].

In the Nigerian context, although few studies on IVF exist, available evidence highlights regional differences in knowledge, perceptions, and attitudes. In the South-South region, studies in the Oredo Local Government Area, Edo State [8], and in the South-East [9] reported low knowledge and acceptance of IVF, as women viewed it as unnatural and costly. Umar & Adamu [10] found that, despite high awareness in Northern Nigeria, IVF was widely misunderstood, leading to hesitancy in use. In the South-West, studies [11, 12] documented adequate knowledge and positive attitudes towards IVF, but affordability remained a major barrier. Generally, despite some technological advances in the health sector, uptake of IVF remains low in Nigeria due to factors such as ignorance, cultural beliefs, religious objections, high costs, and limited access to fertility services [13, 14]. The treatment is shrouded in secrecy and stigma in Nigeria due to misconceptions, religious sentiments, and stigma [1].

Building on these insights, it is important to note that, especially in the South West, where Ekiti State is situated, the available studies were either conducted in tertiary-level care settings or in urban areas where the population has access to adequate information and healthcare services, and where the majority are high-income earners. In contrast, there is a dearth of such studies in rural settings.

To improve maternal health outcomes and address the psychosocial burden of infertility at the grassroots level, it becomes imperative to assess the primary level of care, where exposure to resources is limited, including treatment options for infertility as a result of their geographical location. This study, therefore, aimed to assess the knowledge, perceptions, and attitudes toward in vitro fertilization as a method of conception among women of childbearing age at the grassroots level in Southwestern Nigeria. Specifically, the study focused on the knowledge, perception and attitude of reproductive-aged women regarding IVF as a conception option.

## Literature review

Understanding IVF requires grasping both its historical development and procedural complexity. Early attempts in the 1950s and 1970s laid the groundwork for modern IVF, culminating in the birth of the first “test tube baby”, Louise Brown, in the United Kingdom [15]. Over time, technological advances have led to wider adoption, particularly in developed nations [16]. In Sub-Saharan Africa, a large proportion of infertility cases remain untreated because of limited knowledge and inadequate reproductive health services. Ombelet and Onofre [17] emphasized that fewer than 1.5% of the African population currently access ART services. In Nigeria alone, with a population exceeding 200 million, only about 7,000 IVF cycles were performed in 2022 [14]. These figures highlight the gap between technological availability and public understanding.

Knowledge of IVF is essential not only for uptake but also for its successful application. Unfortunately, misinformation and a lack of counselling continue to undermine utilization, particularly among women of childbearing age. In the Nigerian context, perceptions and attitudes towards IVF are shaped by cultural, religious, educational, and socioeconomic influences (6). While some women view IVF as a breakthrough in reproductive health, others associate it with unnatural conception, religious prohibitions, and social stigma [18, 19]. Moreover, despite their positive attitudes, women in low- and middle-income countries often view IVF as inaccessible because of its high cost, emotional toll, and the limited availability of fertility centers in public health systems [20–22].

## Methods and materials

### Research design and setting

This study employed a descriptive cross-sectional survey design to assess knowledge, perceptions, and attitudes towards in vitro fertilization (IVF) among women of childbearing age. The research was conducted at the three major primary healthcare centers in Ado Ekiti, the capital of Ekiti State in Southwestern Nigeria, namely Okeyinmi Comprehensive Primary Health Care Center, Odo-Ado Basic Health Care Center, and Oke-Oniyo Basic Health Care Center. These facilities provide essential maternal and reproductive health services and were selected for the high volume of women attending antenatal and fertility-related clinics, reflecting diverse experiences and perceptions of IVF. Each center is government-operated and serves a diverse population across socioeconomic and ethnic backgrounds, making it ideal for assessing IVF-related awareness and attitudes in a representative community setting. Moreover, PHC settings often enjoy established community trust, which can facilitate honest responses to sensitive issues such as IVF. The target population comprised women of childbearing age (15–45 years) attending selected primary healthcare centers in Ado Ekiti, Ekiti State, Nigeria. These included women who had either given birth or were within reproductive age and accessing services at Okeyinmi, Odo-Ado, and Oke-Oniyo health centers. The estimated total population across the three health centers over six months was 400 women. Using Taro Yamane’s formula with a 5% margin of error, the calculated sample size was 200. To account for potential non-responses or incomplete questionnaires, a 10% attrition rate was added, yielding a final sample size of 220 participants. Proportional allocation was used to distribute the sample across the three centers, and respondents were selected by simple random sampling using the clinic attendance register as the sampling frame. Selection was done by lottery until the calculated sample size was reached. 83 respondents were selected from both Okeyinmi and Odo-Ado health centers, while 55 were selected from Oke-Oniyo health center, reflecting the patient volume at each facility.

Eligibility criteria included being a woman of reproductive age, attending one of the selected primary healthcare centers in Ado Ekiti during the study period, and willingness to provide informed consent. Participants were also required to be physically and mentally capable of responding to the questionnaire. Women who were critically ill, mentally incapacitated, outside the reproductive age range, or who had previously participated in similar IVF-related surveys, as well as those who declined to give consent, were excluded.

Following ethical approval from the Ekiti State Primary Health Care Board (EK/PHCDA/ADM/316/45), data were collected using a semi-structured, pretested questionnaire adapted from validated tools used in previous IVF-related studies [1]. The instrument consisted of four sections: Section A captured socio-demographic characteristics; Section B assessed knowledge of IVF; Section C examined participants’ perceptions; and Section D evaluated attitudes toward IVF. The questionnaire was reviewed by reproductive health experts for content validity and was pretested among twenty (20) women with similar characteristics outside the study area in PHC, Ago, Ado Ekiti, using the test-retest method in order to ensure reliability and clarity. Cronbach’s alpha coefficient *α* = 0.75.

Data collection spanned 2 months because limited daily attendance necessitated a slower recruitment pace to reach the target sample size. Allowing women sufficient time facilitated adequate coverage of women with varying experiences, preferences, and needs. It also ensured informed consent and respect for the women’s dignity. Literate participants completed the questionnaire independently, whereas a researcher administered the questionnaire to non-literate respondents. Questionnaires were retrieved immediately after completion to ensure completeness and minimize data loss. Confidentiality was maintained throughout the process, and participants were assured that their responses would be used solely for research purposes.

Collected data were coded and entered into IBM SPSS Statistics version 26 for analysis. Descriptive statistics, including frequencies, percentages, means, and standard deviations, were used to summarize demographic characteristics and responses regarding knowledge, perceptions, and attitudes towards IVF. The instrument used was a 4-point Likert scale. The measurement was ordinal, and the distance between ratings cannot be proven mathematically. This allows the use of parametric measures, such as the weighted mean, to assess levels of knowledge, perception, and attitude. Pearson Product-Moment Correlation was employed to examine the relationships between variables at the significance level of *p* < 0.05. Findings were presented using tables for clarity.

## Results

### Demographic characteristics of respondents

Table 1 shows the sociodemographic characteristics of the participants. A total of 222 women of childbearing age participated in the study. The largest age group (63.1%) was aged 20–29 years, while the smallest age group (2.7%) was aged 40 years and above. Most respondents identified as Christians (76.0%), followed by Muslims (23.1%), with only 0.9%] practicing traditional religion. The predominant ethnic group was Yoruba (60.8%), followed by Igbo (19.8%) and Hausa (12.6%). Regarding employment, 55.9% were employed, 27.0% were students, and 17.1% were unemployed.

**Table 1:** Demographic characteristics of the women

Characteristics	Frequency	%
***Age of Participants in Year***		
< 20	15	6.8
20−29	140	63.1
30–39	61	27.5
40–49	6	2.7
***Participants’ Religion***		
Islam	51	23.1
Christianity	168	76.0
Traditional	2	0.9
***Participants’ Tribe***		
Hausa	28	12.6
Igbo	44	19.8
Yoruba	135	60.8
Others	15	6.8
***Participants’ Employment Status***		
Employed	124	55.9
Student	60	27.0
Unemployed	38	17.1

### Knowledge of in vitro fertilization

The information in Table 2 showed the level of knowledge about IVF among women of childbearing age in Ado Ekiti. Most respondents (92.8%) correctly identified the meaning of IVF, and 87.5% agreed that lifestyle factors like smoking and obesity can affect IVF success. However, only 53.2% believed that a woman’s age influences IVF outcomes, suggesting partial gaps in knowledge on age-related fertility decline.

**Table 2:** Frequency counts and mean rating of knowledge level of of IVF among women of childbearing age in Ado Ekiti

Item	*N*	Agree		Disagree		WA	Remark
		F	%	f	%		
IVF means In **v**itro fertilization (IVF)	222	206	92.8	16	7.2	3.39	***High***
The aim of undergoing IVF is to achieve pregnancy	222	192	87.7	27	12.3	3.24	***High***
Lifestyle factors, like smoking and obesity, can affect the success of IVF	222	189	87.5	27	12.5	3.23	***High***
In vitro fertilization is defined as a medical procedure that involves retrieving eggs from a woman’s ovaries and fertilizing them with sperm usually in a laboratory	222	199	89.6	23	10.4	3.23	***High***
IVF is a form of assisted reproductive technology (ART)	222	190	85.6	32	14.4	3.10	***High***
IVF can only be done in a specialized fertility clinic	222	167	75.2	55	24.8	2.89	***High***
A woman’s age has no influence on IVF	222	115	53.2	101	46.8	2.37	***Low***
***Mean of Weighted Average = 3.06***							

***Key:*** N = Number of respondents; f = frequency; % = percentage of respondents; WA = Weighted Average/Mean

To determine the knowledge level of IVF among women of childbearing age in Ado Ekiti (high or low). The low level was determined by scores below the mean cut-off point (*x* < 2.50, i.e., *x* = 1.00-2.49) while the high level was determined by scores above the mean cut-off point (*x* *≥* 2.50, i.e., *x* = 2.50-4.00). Given a weighted mean of 3.06, which falls within the 2.50-4.00 mean cutoff range, the overall knowledge level of IVF among the women was considered high.

### Perception regarding IVF

The information in Table 3 showed that the perception of IVF among women of childbearing age in Ado Ekiti was generally favorable, with a mean weighted average of 2.89. Many participants (84.2%) agreed that IVF offers hope after failed fertility treatments, and 88.3% acknowledged that it allows for biological parenthood. To determine the perception of IVF among the women (favorable or unfavorable), the scores for items 13–21 in section C of the questionnaire were analyzed using frequency counts, percentages, and mean ratings. The unfavorable perception was determined by scores below the mean cut off point (*x* < 2.50 i.e. *x* = 1.00-2.49) while the favorable perception was determined by the score above the mean cut off point (*x* *≥* 2.50 i.e. *x* = 2.50-4.00) Concerns were noted regarding its cost (72.6%) and potential physical/emotional toll (71.1%), but overall perceptions leaned positive.

**Table 3:** Frequency counts and percentage of the perception of IVF among women of childbearing age in Ado Ekiti

Item	*N*	Agree		Disagree		WA	Remark
		F	%	F	%		
IVF offers hope for couples who have tried other fertility treatments without success	222	187	84.2	35	15.8	3.08	***Favourable***
IVF poses significant physical and emotional risks to women	222	155	71.1	63	28.9	2.68	***Favourable***
IVF is a costly and time-consuming process with no guarantee of success	222	156	72.6	59	27.4	2.87	***Favourable***
I think IVF is not accessible	222	159	72.9	59	27.1	2.92	***Favourable***
IVF provides an opportunity for individuals to experience parenthood	222	181	81.5	41	18.5	3.06	***Favourable***
IVF can be a source of stress and anxiety for individuals and couples	222	159	72.9	59	27.1	2.80	***Favourable***
IVF allows individuals to have biological children when other options are not possible	222	185	88.3	37	16.7	3.14	***Favourable***
IVF can affect an individual’s self-esteem and body image	222	144	64.9	77	34.7	2.74	***Favourable***
IVF raises ethical concerns, such as the disposal of unused embryos	222	140	64.8	75	34.7	2.73	***Favourable***
***Mean of Weighted Average = 2.89***							

***Key:*** N = Number of respondents; f = frequency; % = percentage of respondents; WA = Weighted Average/Mean

Given a weighted mean of 2.89, which falls within the 2.50-4.00 mean cutoff range, the overall perception of IVF among the women was considered favorable.

### Attitude toward IVF

Table 4 shows that the women’s attitudes toward IVF were predominantly positive, with a mean score of 3.08. Most participants viewed IVF as morally acceptable (79.7%) and viable for infertility treatment (80.2%). Notably, 87.4% considered the procedure too expensive yet supported its use because of its benefits. To determine the attitude towards IVF among the women (negative or positive), the scores of items 22–26 in section D of the questionnaire were subjected to statistical analysis using frequency counts, percentages, and mean ratings. The negative attitude was determined by scores below the mean cut-off point (*x* < 2.50, i.e., *x* = 1.00-2.49) while the positive attitude was determined by scores above the mean cut-off point (*x* *≥* 2.50, i.e., *x* = 2.50-4.00). Given a weighted mean of 3.08, which falls within the 2.50-4.00 mean cutoff range, the overall attitude towards IVF among women of childbearing age in Ado Ekiti was considered positive.

**Table 4:** Frequency count and percentage of attitude towards IVF among women of childbearing age in Ado Ekiti

Item	*N*	Agree		Disagree		WA	Remark
		F	%	F	%		
IVF is a morally acceptable way to conceive a child	222	177	79.7	45	20.3	2.86	***Positive***
IVF is a viable option for couples struggling with infertility	222	178	80.2	44	19.8	2.96	***Positive***
IVF is too expensive and inaccessible to many people	222	194	87.4	28	12.6	3.31	***Positive***
IVF is a complex and emotionally challenging process	222	190	85.6	32	14.4	3.11	***Positive***
Do you believe that children conceived through IVF can be as healthy as those conceived naturally	222	193	86.9	29	13.1	3.14	***Positive***
***Mean of Weighted Average = 3.08***							

***Key:*** N = Number of respondents; f = frequency; % = percentage of respondents; WA = Weighted Average/Mean

### Hypothesis testing

Two hypotheses were tested using Pearson Product-Moment Correlation to determine the relationship between knowledge and both perception and attitude toward IVF.

- **Hypothesis 1:** There is no significant relationship between knowledge and perception of IVF.
- **Hypothesis 2:** There is no significant relationship between knowledge and attitude toward IVF.

Table 5 shows the relationship between women’s knowledge and their perception of IVF, while Table 6 shows the relationship between their knowledge and attitudes towards IVF. Both null hypotheses were rejected, indicating moderate, statistically significant positive correlations.

**Table 5:** PPMC showing relationship between the knowledge and perception of IVF among women of childbearing age

Variables	*N*	Mean	Std. Dev.	rcal	pvalue
Knowledge of IVF	222	21.25	4.022	0.520*	0.000
Perception of IVF	222	25.71	6.038		

***P < 0.05 (Significant Result)***

**Table 6:** PPMC showing relationship between the knowledge and attitude towards IVF among women of childbearing age

Variables	*N*	Mean	Std. Dev.	rcal	pvalue
Knowledge of IVF	222	21.25	4.022	0.606*	0.000
Attitude towards IVF	222	15.39	3.403		

***P < 0.05 (Significant Result)***

## Discussion

This study assessed the knowledge, perceptions, and attitudes toward in vitro fertilization (IVF) among women of childbearing age at the primary level of care in Ekiti State, Nigeria. Findings revealed a generally high level of awareness and favorable disposition toward IVF, though socioeconomic and accessibility barriers remain influential.

The study found that most respondents demonstrated a high level of knowledge about IVF, particularly regarding its definition, purpose, and procedural elements. This aligns with the findings of Edward et al. [12], who reported high IVF awareness among women in Akure, Nigeria.

However, the finding is at variance with that of Adeyemi [23], who reported low levels of knowledge of IVF among respondents in Lagos, despite it being an urban location where resources abound to enhance people’s awareness and knowledge of IVF. The high level of knowledge observed in this study may be attributed to the smaller clientele in rural settings, which can foster closer relationships with healthcare providers and support open discussions about conception options. Moreover, the providers could be more proactive in discussing IVF. Similarly, Baka et al. [24] observed that while IVF knowledge was moderate in Riyadh, it significantly correlated with age and marital status. However, a slight knowledge gap was noted in this study regarding the impact of age on IVF success; this is consistent with Arhin et al. [25], who documented limited awareness of IVF risk factors and determinants of success in Hungary. These findings support the role of education and reproductive health literacy in promoting IVF utilization.;

Respondents expressed favorable perceptions of IVF, acknowledging its ability to offer hope and facilitate biological parenthood. This is consistent with Szalma et al. [26], who found that 81.7% of women in Ghana accepted IVF, despite concerns about affordability and naturalness. Similarly, Xu et al. [27] emphasized that knowledge significantly shapes perceptions and reduces anxiety about IVF. Yet, perceptions were tempered by concerns about cost, ethical issues, emotional tolls and accessibility which were also observed in previous studies [28, 29] which can hinder IVF’s full acceptance,

The study revealed a predominantly positive attitude, with most women considering IVF morally acceptable and a viable option for managing infertility. This is in line with Joe – Ikechebelu et al. [1], who found strong support for ART use among Saudi women. Appiah and Ganle [30] also reported similar patterns in sub-Saharan Africa. However, affordability remained a key challenge, echoing findings from Njagi et al. (20), who noted that IVF costs often exceed average incomes in low- and middle-income countries. Additionally, occupational and emotional stressors were found to impact women’s willingness to pursue IVF, a factor supported by Leigh and Skuthan [31].

Overall, this study reaffirms that knowledge significantly influences both perception and attitude, as confirmed by the statistically significant correlations found in the analysis. These findings underscore the need for targeted reproductive health education, psychosocial support, and economic interventions to improve IVF access and acceptance among Nigerian women of childbearing age. It is also expedient that primary healthcare workers, especially nurses/midwives, integrate IVF education into antenatal and family planning services, explain concepts to clients in simple language to dispel myths and misconceptions that could cause emotional stress. At the facility level, basic identification of infertility could be made during history taking, which could be followed up by counselling and prompt referral to specialists for further investigation and medical attention.

## Limitation of study

This study has a few limitations that should be considered when interpreting the findings. First, the research was limited to three selected primary healthcare centers in Ado Ekiti, which may restrict the generalizability of the results to other regions or rural populations with different sociocultural and economic dynamics. Second, though all the settings involved are primary health care facilities, they are all located in Ado – Ekiti. This reflects urban bias. Third, data were collected using self-reported questionnaires, which may be subject to response bias, including social desirability and recall errors. Fourth, the cross-sectional design captures perceptions and attitudes at a single point in time, limiting the ability to assess changes over time or causal relationships. Also, the study lacked differentiation among respondents’ socio-demographics, knowledge, and attitudes towards IVF, which limits the depth of explanation in this regard. Despite these limitations, the study offers valuable insights into women’s knowledge, perceptions, and attitudes toward IVF and can inform future reproductive health interventions in similar contexts.

## Implications for primary health care practice

The findings of this study have important implications for primary health care practice, particularly in reproductive health education, counselling, and patient advocacy. Nurses, as frontline health professionals, play a pivotal role in raising awareness and correcting misconceptions about in vitro fertilization (IVF) among women of childbearing age. Given the high level of knowledge observed, yet persistent concerns about accessibility and emotional burden, nurses must be equipped to provide accurate information, emotional support, and culturally sensitive guidance throughout the fertility care continuum. Moreover, integrating IVF education into routine maternal and child health services can enhance early decision-making and reduce stigma. Nurses are also well-positioned to advocate for policies that improve access to fertility services and reduce financial and psychological barriers.

At the regional level, increased access to fertility services is essential. This may be achieved by budgeting for IVF services, establishing more centers, subsidizing costs, and forming public-private partnerships. There could also be sponsored/targeted programs to address awareness, knowledge gaps, and cultural misconceptions. Ultimately, empowering nurses/midwives with training and resources on assisted reproductive technologies will improve client outcomes and support reproductive autonomy.

## Conclusion

This study assessed the knowledge, perceptions, and attitudes toward in vitro fertilization (IVF) among women of childbearing age in Ado Ekiti, Nigeria. The findings revealed that a majority of the participants had a high level of knowledge about IVF, coupled with favorable perceptions and positive attitudes toward its use as a method of conception. However, concerns such as the cost, accessibility, and emotional implications of IVF were notable among respondents. Statistical analysis confirmed moderately significant correlations between knowledge and both perception and attitude, emphasizing the critical role of awareness in shaping reproductive health choices. These results underscore the need for enhanced educational and policy efforts to improve access to and acceptance of IVF, particularly among women in low- and middle-income settings. Promoting accurate information, reducing stigma, and improving affordability are essential steps toward supporting the reproductive rights and health of women seeking fertility solutions.

## Data Availability

All data generated or analysed during this study are included in this published article.
